# Supplementary material for: Monitoring the Evolution of the Aroma Profile of Lager Beer in Aluminium Cans and Glass Bottles during the Natural Ageing Process by Means of HS-SPME/GC-MS and Multivariate Analysis
Source: Molecules. 2023 Mar 20;28(6):2807. doi: 10.3390/molecules28062807 (PMC10055024; doi:10.3390/molecules28062807)
Supplement: Supplementary file 1 [file molecules-28-02807-s001.zip › molecules-2253712-supplementary.pdf]

Supplementary Material

1. Figure S1

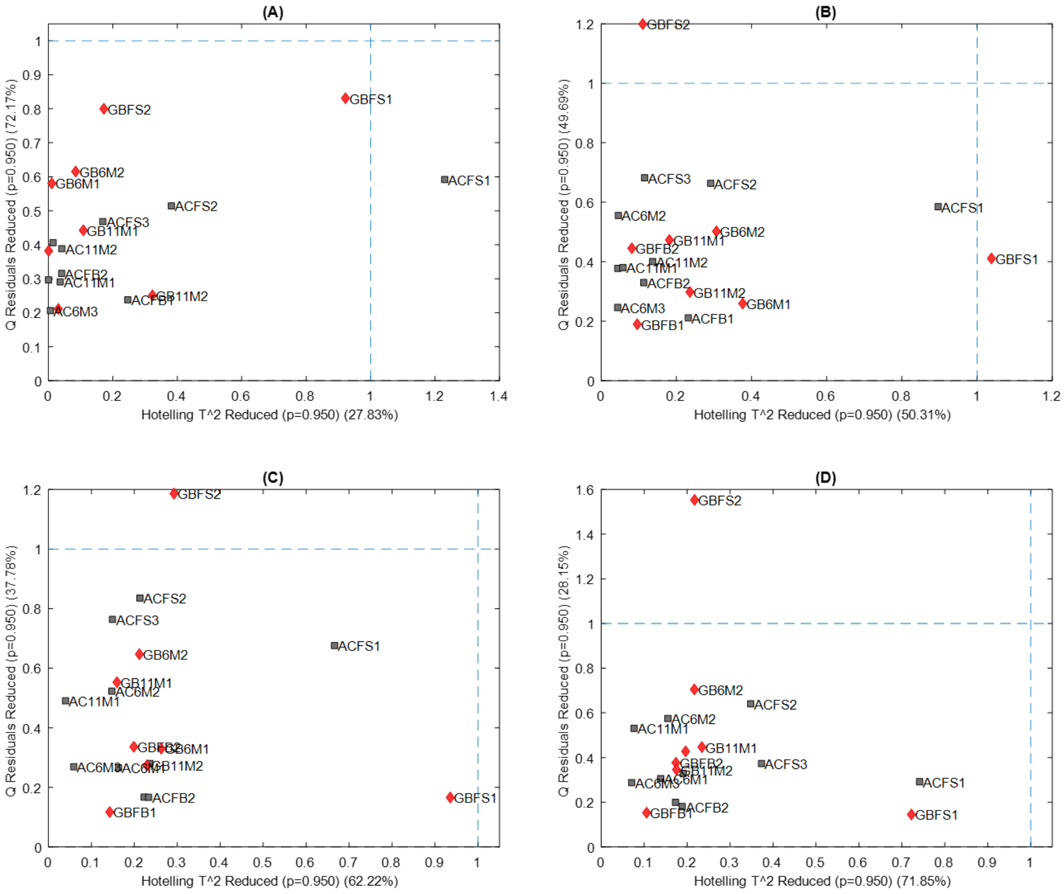

Figure S1: Hotelling T^2 vs Q residual plots for the PCA models with (A) one, (B) two, (C) three, and (D) four principal components.
